# Venous waterfalls mainly buffer backward pressure transmission

**DOI:** 10.1186/s40635-026-00889-2

**Published:** 2026-04-16

**Authors:** Ricardo Castro, Eduardo Kattan, Jaime Retamal, Glenn Hernández, Michael R. Pinsky

**Affiliations:** 1 Departamento de Medicina Intensiva, Facultad de Medicina, Pontificia Universidad Católica de Chile, Av. Diagonal Paraguay #362 Piso 6, 8330049 Santiago Centro, RM Chile; 2 Hospital Clínico UC-CHRISTUS, Pontificia Universidad Católica de Chile, Santiago, Chile; 3 Department of Critical Care Medicine, University of Pittsburgh, Pittsburgh, PA USA

**Dear Editor,**

We thank Kenny and Moller for their thoughtful correspondence regarding our review [1]. We agree with their central hydraulic point: any Starling-resistor element implies a collapsible segment that can become flow-limiting, and relative to a fully patent conduit, may reduce organ venous drainage. Their analogy with expiratory flow limitation appropriately emphasizes that waterfall behavior should not be interpreted as intrinsically beneficial for venous outflow.

A key clarification is that our use of *protection* was not intended to imply enhanced venous drainage under all conditions. Rather, we used the term in the narrower sense of buffering backward pressure transmission from downstream venous pressures to the microcirculation. In a venous waterfall regime, rises in right atrial pressure (Pra) are not necessarily transmitted linearly to small venules and capillaries until a local threshold is exceeded. This thresholded transmission may delay capillary hypertension, edema formation, and pressure-dependent microvascular injury, and may contribute to territory-specific vulnerability. In this view, the relevant endpoint is not maximal steady venous outflow, but the degree and timing with which downstream pressures reach the capillary bed.

We also agree with Kenny and Moller that waterfall behavior is dynamic and volume-state dependent, with “zone transitions” analogous to West zones as downstream pressures change. However, their argument largely rests on a steady-state, single-segment geometric Starling-resistor model. While hydraulically correct, this static representation is incomplete for critical illness, where venous outflow is distributed across venules and small veins embedded in deformable parenchyma, and where external/interstitial pressures, compliance, and recruitment are state- and time-dependent (e.g., edema, capsular constraint, abdominal pressure, and ventilatory pressures) [2]. Clinically, this dynamic behavior is evident during transient rises in Pra (e.g., cough or Valsalva), which may be buffered when a venous waterfall is operative; similarly, spontaneous inspiration with diaphragmatic descent can transiently increase intrahepatic interstitial pressure, potentially augmenting an intrahepatic venous waterfall [3]. Consistent with this concept, pressure-flow analysis in porcine liver demonstrates an effective backpressure exceeding hepatic venous pressure in both hepatic artery and portal vein.

The observations cited by Kenny and Moller, showing that increasing Pra during inspiratory holds may reduce hepatosplanchnic venous Starling resistors and increase hepatic outflow in euvolemia, fit within this framework. They do not negate buffering; rather, they illustrate that abolishing a waterfall can increase outflow under specific operating conditions. Conversely, in fluid overload, high Pmsf and elevated venous pressures may favor direct upstream pressure transmission and outflow limitation, increasing the likelihood of microcirculatory congestion.

Finally, we concur that this state dependence is one reason inspiratory-hold maneuvers and are problematic for estimating Pmsf. Inspiratory holds raise Pra but also alter intrathoracic/abdominal pressures, thereby changing collapsibility and effective critical pressures; if the maneuver modifies the venous return function itself, extrapolation to a single patient-invariant Pmsf becomes unreliable. We emphasize that our postulate was conceptual and mechanistic, aimed at organizing venous congestion physiology, rather than to advocate a specific bedside estimator.

Ricardo Castro

Eduardo Kattan

Jaime Retamal

Glenn Hernández

Michael R Pinsky

## Data Availability

Not applicable. This article does not include original datasets; all information are derived from previously published sources cited in the reference list.
